# Psychometrics: Applications in Nursing

**DOI:** 10.1590/1518-8345.0000.3992

**Published:** 2023-08-04

**Authors:** Renata Eloah de Lucena Ferretti-Rebustini

**Affiliations:** 1 Universidade de São Paulo, Escola de Enfermagem, Departamento de Enfermagem Médico-Cirúrgica, São Paulo, SP, Brasil

**Figure:**
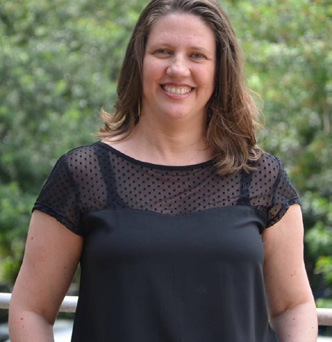


The phenomena evaluated by nurses in their professional or scientific practice have become complex and require multiplicity of diverse information for the formation of critical thinking and clinical reasoning. The health evaluation paradigms have changed, imposing the need for an evolution in the evaluation processes and methods, for measuring both objective and subjective phenomena.

Evaluating subjective and indirectly observable phenomena can be a challenge, as it depends on an objective measurement that serves as evidence to strengthen nurses’ clinical impression during the assessment. Measuring instruments (tests, scales, inventories and indices, among others)^(^(1)^)^ render measurements objective. In this way, such instruments can be used to detect, quantify, describe, inform, explain and predict different phenomena, in addition to guiding courses of action.

In Nursing, instruments support the teaching and learning process in nurses’ initial, continuing and permanent training; they subsidize the clinical evaluation inherent to the Nursing Process and advanced practice; and they direct care management and measure phenomena in scientific research studies. However, for them to fulfill their measuring role, they should have their metric properties analyzed.

The measuring science must be employed to analyze such properties. Psychometrics is concerned with measuring and predicting psychological, attitudinal and behavioral phenomena^(^(2)^)^. It can also be used from a clinimetric perspective. When understood as a research method, it is concerned with evaluating the attributes of an instrument with regard to the type of information generated, data validity and reliability, relating to the procedures used to evaluate such attributes^(^(1)^)^, but it is not restricted to using statistical techniques to analyze data related to the application of instruments^(^(3)^)^. It uses its own theoretical framework to ground and direct the way to analyze the data, in a quantitative and qualitative combined perspective related to the intended type of evidence.

Psychometrics is guided by two theoretical models: the Classical Test Theory (CTT) and the Item Response Theory (IRT)^(^(1)^)^. Each of these models will direct the application of psychometric procedures from different perspectives. CTT addresses an instrument from the perspective of the set of items that comprise it and IRT does so from the perspective of each item individually^(^(1)^)^. In both models, a set of procedures and combined techniques assess the validity and reliability evidence, which in turn represent the necessary attributes to demonstrate the metric quality of an instrument.

Validity and reliability are different but interrelated attributes. Validity represents to which extent an instrument evaluates what it is intended to measure, from different perspectives related to the type of validity under analysis, being considered the most important evidence of the metric quality of an instrument^(^(1)^)^. Without its analysis, measurements are potentially dangerous, especially when they support courses of action in health. Reliability relates to constancy of the assessment product at different times or situations^(^(1)^)^. Both should be based on solid empirical evidence.

In fact, the psychometric science has evolved in such a way that, currently, its theoretical models are understood as complementary and equally important. They differ in the analysis objective and in the type of inference to be presented. What contemporary Psychometrics requires is the use of combined procedures for data analysis and the validity types have also been changing in this evolution. Currently, there are five types of validity evidence (test content, response process, internal structure, association with other variables, and consequences of use)^(^(1)^)^, each with a set of specific techniques to be employed in a chained fashion. Techniques were improved, new indices were incorporated and software were developed^(^(1)^,^(4)^)^. The major challenge for researchers is to know when to use one technique or another, according to the analysis objective and to the theoretical model that should be based on the guiding principles of Psychometric science^(^(4)^)^. There is no more room for using techniques that have proved to be outdated for decades^(^(5)^)^. Employing outdated procedures violates the principles of integrity and good scientific practices and represents a disservice to society, in addition to constituting a major ethical problem^(^(1)^,^(5)^)^.

Herein understood here as a measuring science and not merely as a set of statistical techniques used to analyze instrument evaluation data, Psychometrics has much to contribute to Nursing. It can be used to create, adapt and/or analyze diverse validity and reliability evidence of instruments that support nurses’ scientific and professional practice. As it is based on a contemporary and well-applied theoretical framework, it contributes to the design of studies with high methodological rigor and to the development of Nursing knowledge.

The availability of measuring instruments that really evaluate the phenomena of interest in the Nursing and Health areas with validity and precision is essential for care quality and patient safety. It is the duty of all nurses to ground their professional practice on the best evidence available. It is the duty of all researchers to produce knowledge derived from research guided by the best scientific practices. More than a scientific duty, it is a social obligation.

**References**
